# Supplementary figures and images for: Unveiling immune cell response disparities in human primary cancer-associated fibroblasts between two- and three-dimensional cultures
Source: PLoS One. 2024 Dec 19;19(12):e0314227. doi: 10.1371/journal.pone.0314227 (PMC11658583; doi:10.1371/journal.pone.0314227)

## Slide 1
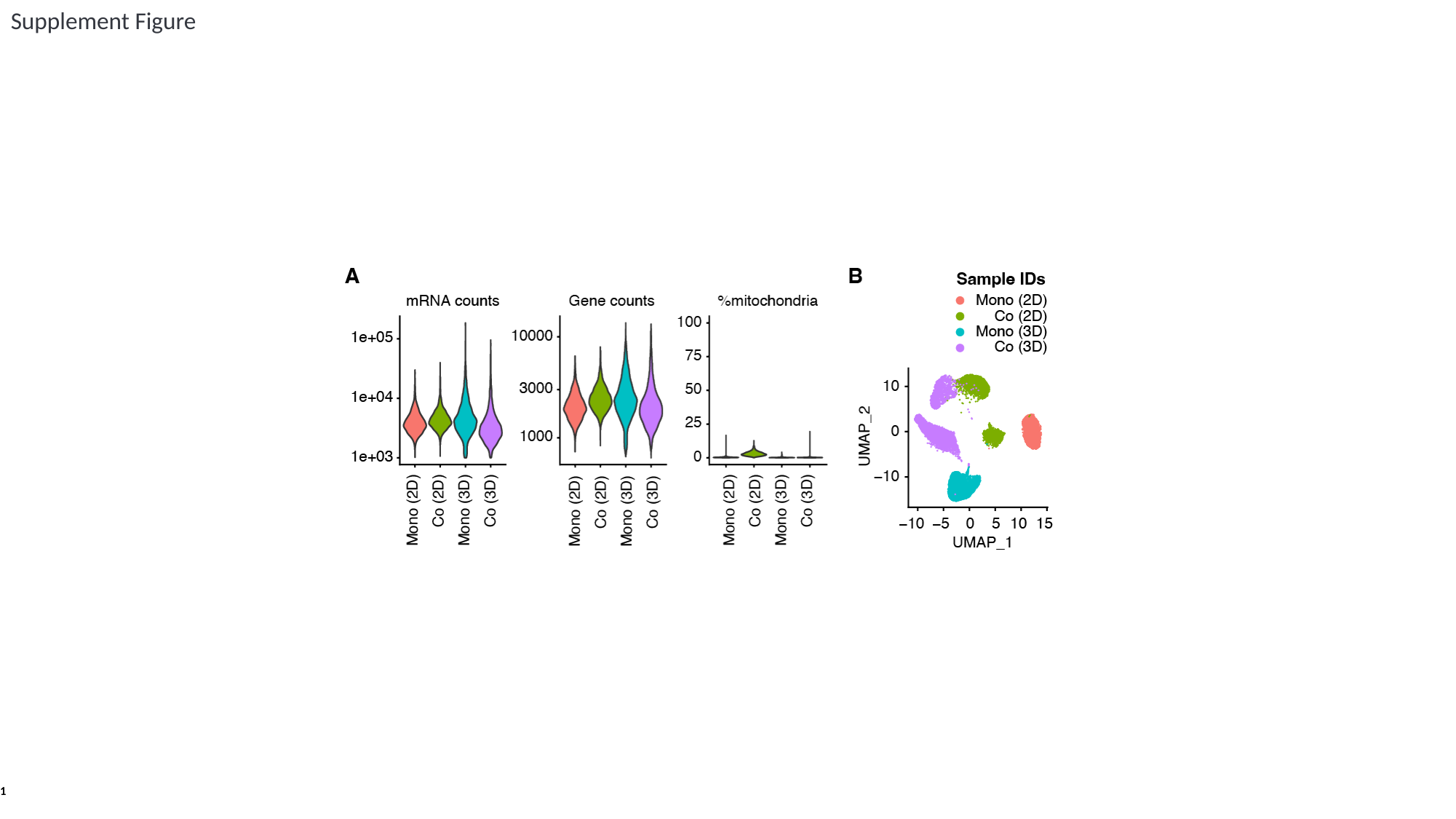

Supplement Figure
1

Supplement: S1 Fig — (A) Violin plots displaying the QC metrics of the snRNA-seq libraries used in this study after quality control filtering. The distribution of mRNA molecular counts, gene counts, and the percentage of mitochondrial mRNAs within individual libraries are illustrated in the left, middle, and right panels, respectively. (B) Two-dimensional UMAP visualization displays the clustering of 16,235 single-nuclei transcriptomes produced in this study, colored according to the cell culture conditions. Abbreviations: Mono, monoculture of cancer-associated fibroblasts (CAFs); Co, co-culture of CAFs with BxPC3 pancreatic cancer cells; 2D, two-dimensional monolayer culture; 3D, three-dimensional spheroid cell culture. (PPTX) [file pone.0314227.s001.pptx]
