# Supplementary material for: Unveiling immune cell response disparities in human primary cancer-associated fibroblasts between two- and three-dimensional cultures
Source: PLoS One. 2024 Dec 19;19(12):e0314227. doi: 10.1371/journal.pone.0314227 (PMC11658583; doi:10.1371/journal.pone.0314227)
Supplement: S1 Table — (DOCX) [file pone.0314227.s002.docx]

**S1 Table. The panel of macrophage-specific markers for flow cytometry analysis:**

| Marker | Flurophore | Clone | Manufacturer | Catalog | Dilution |
| --- | --- | --- | --- | --- | --- |
| CD68 | Alexa Fluor 488 | Y1/82A | BioLegend [877-246-5343] | 333812 | 1/100 |
| VSIG4 | APC | JAV4 | Thermo Fisher Scientific [1 800 955 6288] | 17-5757-42 | 1/100 |
| HLA DR | Alexa Fluor 700 | LN3 | BioLegend [877-246-5343] | 327014 | 1/300 |
| CD14 | APC-H7 | MphiP9 | BD Biosciences [877.232.8995] | 560180 | 1/100 |
| CD86 | Brilliant Violet 421 | 2331 (FUN-1) | BD Biosciences [877.232.8995] | 562432 | 1/100 |
| Fixable Live/Dead | Zombie Aqua |  | Biolegend | 423102 | 1/1000 |
| CD33 | Brilliant Violet 605 | P67.6 | BioLegend [877-246-5343] | 366612 | 1/100 |
| CD80 | Brilliant Violet 711 | 2D10 | BioLegend [877-246-5343] | 305236 | 1/100 |
| CD11b | Brilliant Violet 650 | M1/70 | BD Biosciences [877.232.8995] | 563402 | 1/500 |
| CD163 | Brilliant Violet 785 | GHI/61 | BioLegend [877-246-5343] | 333632 | 1/100 |
| CD206 | PE | 15-2 | BioLegend [877-246-5343] | 321106 | 1/100 |
| CD45 | PE-Cy5.5 | HI30 | Thermo Fisher Scientific [1 800 955 6288] | MHCD4518 | 1/100 |
