# Supplementary material for: Unveiling immune cell response disparities in human primary cancer-associated fibroblasts between two- and three-dimensional cultures
Source: PLoS One. 2024 Dec 19;19(12):e0314227. doi: 10.1371/journal.pone.0314227 (PMC11658583; doi:10.1371/journal.pone.0314227)
Supplement: S2 Table — (DOCX) [file pone.0314227.s003.docx]

**S2 Table。IgG controls for flow cytometry analysis:**

| **Fluorophore** | **Isotype** | **Manufacturer** | **Catalog** | **Dilution** |
| --- | --- | --- | --- | --- |
| Alexa Fluor 488 | Mouse IgG 2b,k | BioLegend | 400329 | 1/100 |
| APC | Mouse IgG 2a kappa | eBioscience | 17-4724-81 | 1/100 |
| Alexa Fluor 700 | Mouse IgG2b, κ | Biolegend | 400334 | 1/300 |
| APC-H7 | Mouse IgG2v,k | BD Biosciences | 560183 | 1/100 |
| Brilliant Violet 421 | Mouse IgG1,k | Bd horizon | 562438 | 1/100 |
| Brilliant Violet 605 | Mouse IgG1,k | biolegend | 400162 | 1/100 |
| Brilliant Violet 711 | Mouse IgG1,k | biolegend | 400168 | 1/100 |
| Brilliant Violet 650 | Mouse IgG 2b, kappa | BD Biosciences | 563437 | 1/500 |
| Brilliant Violet 785 | Mouse IgG1, k | biolegend | 400170 | 1/100 |
| PE | Mouse IgG1,k | biolegend | 400112 | 1/100 |
| PE-Cy5.5 | Mouse IgG1 kappa | Life Technologies | 35-4714-82 | 1/100 |
